# Effect of the flavonoid compound glabridin on tachyzoites and bradyzoites of *Toxoplasma gondii*

**DOI:** 10.1186/s13071-025-06695-1

**Published:** 2025-02-17

**Authors:** Yanhua Qiu, Weiwei Wang, Qing Wang, Hongling Lin, Yubin Bai, Jiyu Zhang

**Affiliations:** 1 Key Laboratory of New Animal Drug Project of Gansu Province, Lanzhou, China; 2 Key Laboratory of Veterinary Pharmaceutical Development, Ministry of Agriculture and Rural Affairs, Lanzhou, China; 3 Lanzhou Institute of Husbandry and Pharmaceutical Sciences, Chinese Academy of Agricultural Sciences, Lanzhou, China

**Keywords:** *Toxoplasma gondii*, Flavonoid compound, Glabridin, Autophagy, Mitochondrial dysfunction, Membrane disruption

## Abstract

**Background:**

*Toxoplasma gondii* (*T. gondii*) is one of the most prevalent parasites worldwide. At present, the majority of drugs used for the treatment of toxoplasmosis target the tachyzoite stage of *T. gondii* and are largely ineffective against bradyzoites. Furthermore, these treatments are typically accompanied by adverse events. Consequently, there is an urgent need for the development of novel drugs that are both safe and effective against *T. gondii*.

**Methods:**

A total of 20 flavonoids were preliminarily screened for their anti-*T. gondii* activity using microscopy. Next, the cell counting kit (CCK)-8 method was employed to assess the toxicity of glabridin (GLA) to host cells, while the RH strain of *T.0gondii*, which expresses β-galactosidase, was utilized to evaluate the inhibitory, anti-invasive, and antiproliferative effects of GLA on *T. gondii*. In addition, the Prugniaud (PRU) strain was employed to investigate the impact of GLA on the bradyzoites of *T. gondii*. Subsequently, the effect of GLA on the ultrastructure of *T. gondii* was examined via transmission electron microscopy (TEM), followed by an assessment of the influence of GLA on the autophagy and mitochondria of *T. gondii* through monodansylcadaverine (MDC), MitoTracker^™^ red CMXRos, and CM-HDCFDA and MitoSOX Red staining.

**Results:**

Among the 20 flavonoids assessed, GLA exhibited the most potent anti-*T. gondii* activity. Indeed, it significantly inhibited both the invasive and proliferative abilities of *T. gondii*, thereby disrupting its lytic cycle. Moreover, GLA markedly reduced the number of bradyzoites and concurrently inhibited cyst growth. Meanwhile, ultrastructural analysis revealed that GLA induced mitochondrial swelling, membrane rupture, and autophagy in *T. gondii*. Finally, fluorescent probe staining provided further evidence that GLA triggers mitochondrial dysfunction and autophagy in this parasite.

**Conclusions:**

Our findings collectively indicate that the flavonoid compound GLA exhibits significant activity against both *T. gondii* tachyzoites and bradyzoites. The underlying mechanism of action potentially involves the induction of autophagy and mitochondrial dysfunction and the disruption of the membrane of *T. gondii*, thereby offering new avenues for treating toxoplasmosis and establishing a theoretical reference for future research.

**Graphical Abstract:**

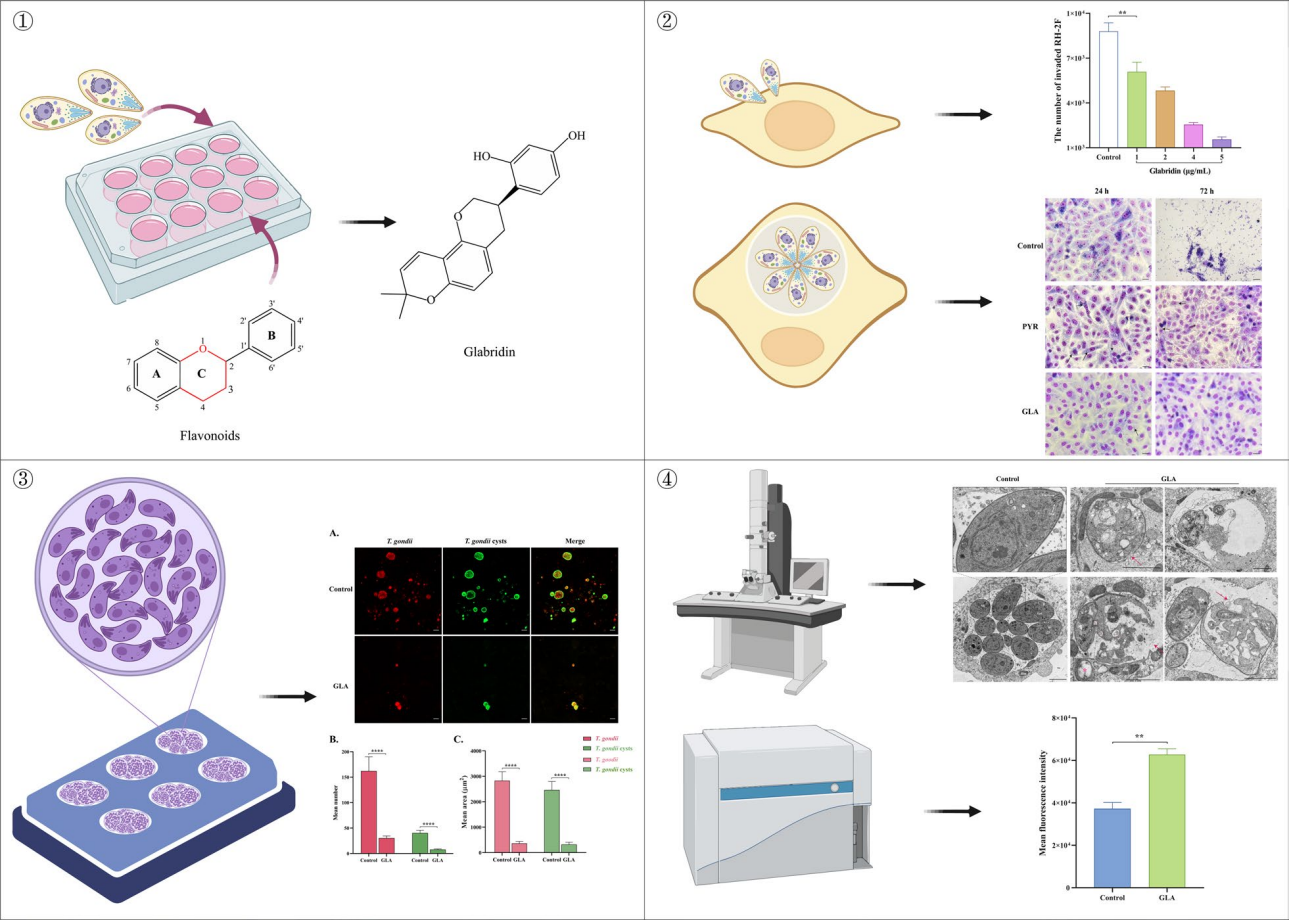

## Background

*T. gondii* is an obligate intracellular parasitic protozoan with a diverse range of hosts, capable of infecting nearly all warm-blooded animals, as well as some mollusks and cold-blooded animals [1, 2]. Notably, approximately one-third of the global population is infected with *T. gondii* [3]. While *T. gondii* infection in immunocompetent individuals is generally mild or asymptomatic, this parasite can evade the host’s innate immune responses, leading to chronic infection [4, 5]. More importantly, *T. gondii* infection can pose life-threatening risks in individuals with compromised immune systems [6]. A growing body of evidence suggests that latent infection with *T. gondii* is associated with various central nervous system disorders [7], including bipolar disorder [8], schizophrenia [9], and autism [10].

At present, the gold-standard treatment for toxoplasmosis remains the combination of sulfadiazine and pyrimethamine (PYR) [11]. While this treatment regimen effectively addresses acute infections caused by *T. gondii* tachyzoites, it does not affect the bradyzoites and cysts of *T. gondii*. Furthermore, this treatment is associated with numerous side effects, including blood toxicity and bone marrow suppression [12]. Alternative treatment modalities, such as azithromycin, atovaquone, and clarithromycin, similarly fail to eradicate *T. gondii*-associated chronic infections [3]. Consequently, there is a pressing need to identify safe and effective drugs that specifically target *T. gondii* bradyzoites and cysts for the treatment of toxoplasmosis.

Natural products have historically played a crucial role in drug discovery and development, serving as a primary source of new pharmaceuticals [13]. They are also essential for identifying new treatments for *T. gondii*. Flavonoids, a popular class of natural products, are classified as plant secondary metabolites with a polyphenolic structure and are widely distributed among various plant species [14]. Currently, numerous flavonoids, including quercetin [15], licochalcone A [16], and myrislignan [17], have demonstrated activity against *T. gondii*. Consequently, this study selected 20 flavonoids to screen their anti-*T. gondii* activity and conducted a series of in vitro studies on GLA, which exhibited the most potent anti-*T. gondii* activity.

## Methods

### Cell and parasites

African green monkey kidney (Vero) cells and human foreskin fibroblasts (HFF) were procured from the cell bank of the Chinese Academy of Sciences. *T. gondii* RH tachyzoites expressing β-galactosidasde (RH-2F) were purchased from the ATCC (article number: 50839). The tachyzoites of *T. gondii* type I strain RH and type II strain PRU were acquired from the Lanzhou Veterinary Research Institute of the Chinese Academy of Agricultural Sciences. Among them, RH-2F is a *T. gondii* RH strain that expresses β-galactosidase, while type I strain RH is a virulent strain that typically induces acute infection in mice, resulting in a mortality rate of 100% [18], and type II PRU is a moderately virulent strain with relatively weak virulence. Mice generally develop cysts in tissues following acute infection, which progress to chronic infection [19].

Vero and HFF cells were cultured in a Dulbecco’s modified eagle medium (DMEM; Gibco) supplemented with 10% fetal bovine serum (FBS; Gibco), 1% nonessential amino acids (Gibco), 1% Glutamax (Gibco), and 1% Sodium pyruvate (Gibco). Parasites were grown and passaged in HFF or Vero cells using DMEM supplemented with 3% FBS. When most of the tachyzoites had lysed the cells, the cells were harvested using a cell scraper and aspirated twice using a 27G needle. After centrifugation at 200 g for 5 min, the supernatant was filtered through a 3 μm pore filter using a syringe with a 27G needle and subsequently centrifuged at 1500 g for 10 min to isolate purified tachyzoites. Lastly, the tachyzoites were resuspended in a culture medium, stained with trypan blue, and counted using a blood cell counting plate for subsequent experiments and passages.

### Screening of anti- *T. gondii* activity of flavonoids

A total of 20 flavonoids (MCE) (Table 1) were dissolved in dimethyl sulfoxide (DMSO; MCE) to prepare a 20 mg/mL stock solution. RH tachyzoites (3 × 10^5^/mL) were inoculated into 12-well plates containing a monolayer of Vero cells. After 8 h, the test groups were incubated with predefined compounds at high (40 μg/mL) and low (5 μg/mL) concentrations in the medium. The control group was only infected with RH without any additional treatment. The wells containing only cells and medium were used as the blank control group. Three replicate wells were established for each group and observed under an inverted microscope at 24, 48, and 72 h, respectively. In addition, we counted the number of parasites in five randomly selected fields in each well and compared them with the control group. The active compounds were screened on the basis of the criterion that the number of *T. gondii* in the field of view was reduced by more than 80% compared with the control group.

**Table 1:** Screening results of anti-*T. gondii* activity of 20 flavonoids

Serial number	Compound name	High concentration (40 μg/mL)	Low concentration (5 μg/mL)
1	Epicatechin	−^a^	−
2	Epigallocatechin	−	−
3	Quercitrin	−	−
4	Isoorientin	−	−
5	Isorhamnetin	+ (cytotoxic)^b^	−
6	Vitexin	−	−
7	Catechin hydrate	−	−
8	Apigenin	+ (cytotoxic)	+ (cytotoxic)
9	Quercetin 3-β-d-glucoside	−	−
10	Azaleatin	−	−
11	Astragalin	−	−
12	Isohyperoside	−	−
13	Avicularin	−	−
14	Galangin	+ (cytotoxic)	−
15	α-Mangostin	Cytotoxic^c^	Cytotoxic
16	Glabridin	Cytotoxic	+ ^d^
17	Silibinin	+ (cytotoxic)	−
18	Farrerol	−	−
19	Scullcapflavone II	−	−
20	Loureirin B	−	−

^a^Ineffective

^b^Effective against *T. gondii*; however, it also exhibits toxicity to host cells

^c^Demonstrates significant toxicity to host cells, resulting in a substantial loss of basic cellular structures

^d^Effective

### Cytotoxicity assay

The toxicity of GLA to Vero cells was determined using the cell counting kit-8 (CCK-8; MCE) method. Briefly, Vero cells (1 × 10^5^ cells/mL) were seeded in 96-well plates and cultured for 12 h. Following this, various concentrations of GLA (16, 12, 10, 8, 6, 5, 4, 2, and 1 μg/mL) were added. The control group consisted of cells and medium, while the blank control contained only medium to account for background interference. CCK-8 reagent was introduced after 72 h of culture, and the absorbance of each well was measured at a wavelength of 450 nm after 1 h of incubation.

### Inhibition assay

RH-2F tachyzoites expressing β-galactosidase were utilized for the growth inhibition assay. Fresh RH-2F tachyzoites were harvested from Vero or HFF cells, and their concentration was adjusted to 2 × 10^3^/mL. Different concentrations of GLA were prepared, with 0.1% DMSO serving as the control group and 5 μg/mL PYR (MCE) as the positive control. The compounds were added to a monolayer of Vero cells in a 96-well plate. After 72 h of incubation, 10 μL of 10% Triton X-100 (Sigma) and 1 mM chlorophenol red-β-D-galactopyranoside (CPRG, Sigma) were introduced into each well, and the resulting mixture was incubated for an additional 12 h in an incubator, and absorbance was measured at 570 nm.

### Proliferation assay

The Giemsa staining test was initially performed by adding 2 × 10^5^ RH tachyzoites to each well containing the monolayer of Vero cells in the 6-well plate. After 2 h, uninvaded tachyzoites were discarded via washing, followed by the addition of 5 μg/mL of GLA. The negative control group contained 0.1% DMSO, and the positive control group consisted of 5 μg/mL PYR. The cells were fixed with cold methanol (Sigma) at 24 h and 72 h, respectively. After washing with phosphate buffer solution (PBS; Solarbio), giemsa staining solution (Sigma) was applied for 15 min. Subsequently, the cells and parasites were visualized and photographed under an inverted microscope.

To further quantify the antiproliferative effect of GLA on *T. gondii*, 1 × 10^4^ RH-2F tachyzoites were added to each well of Vero cells in 96-well plates. After 2 h, the extracellular tachyzoites were removed via washing, and the compounds were added. At 24 h and 72 h, a standard curve was established by adding a known quantity of tachyzoites diluted in a gradient to a well containing only cells. Afterward, Triton X-100 and CPRG were added, and absorbance was measured at a wavelength of 570 nm. The number of parasites in each group of wells was determined on the basis of the standard curve.

### Invasion assay

The 1, 2, 4, and 5 μg/mL GLA solutions were prepared using 5 × 10^5^/mL RH-2F tachyzoites, with a 0.1% DMSO solution used as the control group. After pretreatment at 37 °C for 1 h, the solutions were added to a 96-well plate containing Vero cells. After 2 h, noninvasive tachyzoites were washed with PBS, added to the medium, and the standard curve was established. Similar to the proliferation assay, absorbance was subsequently measured, and the number of tachyzoites invading cells in each well was quantified on the basis of a standard curve.

### Plaque assay

As previously described [20], 300 RH tachyzoites were introduced into a 6-well plate containing a monolayer of HFF cells, followed by a 2-h incubation period to facilitate tachyzoite invasion. Next, 5 μg/mL GLA and 0.1% DMSO were added to the wells. After 7 days of incubation, the cells were fixed with cold methanol for 15 min, washed with PBS, and stained with 1% crystal violet (Sigma) for an additional 15 min. Finally, the excess dye was removed by washing with PBS, and images were captured on a luminous white plate.

### Bradyzoite assay

The 1 × 10^6^ PRU tachyzoites were added to Vero cells in 6-well glass plates. After 2 h of invasion, the tachyzoites were washed with PBS, and the medium was replaced with differentiation medium (1% FBS and 50 mM HEPES (Thermo Fisher), pH 8.2), and cells were transferred to a 37 °C incubator containing 0% CO_2_. The medium was replaced approximately every 12 h. After 7 days, 5 μg/mL of GLA was added, with 0.1% DMSO acting as the negative control group. The differentiation medium, containing various concentrations of compounds, was changed every 12 h. After 7 days of incubation, the cells were fixed with recently prepared 4% formaldehyde solution (Solarbio), permeabilized with 0.2% Triton X-100 for 30 min, blocked with 10% goat serum (Solarbio) for 30 min, and then incubated with anti-*T. gondii* antibody (Abcam, ab138698) at 4 °C overnight. Next, the cells were incubated with the goat anti-mouse Alexa Fluor 647 secondary antibody (Abcam; ab150115) for 2 h at room temperature and then washed with PBS before staining with 10 μg/mL FITC-conjugated Dolichos biflorus agglutinin (FITC-DBA; Thermo Fisher) for 30 min. Finally, they were observed and photographed under a laser confocal microscope (LSM 800, Carl Zeiss, Germany).

### Ultrastructure analysis

A total of 8 × 10^6^ RH tachyzoites were added to Vero cells in a 75 cm^2^ cell culture flask. After 12 h, 5 μg/mL of GLA was added, while the control group received 0.1% DMSO. After 24 h of incubation, the liquid in the cell culture bottle was discarded. After washing twice with PBS, the cells were subjected to trypsin digestion. Afterward, they were centrifuged. After washing twice with PBS, cells and parasite deposits were prefixed with 2.5% glutaraldehyde (Sigma) at room temperature for 30 min and then transferred to a 4 °C refrigerator overnight. The sample was pre-embedded in 1% agarose (Sigma) and fixed with 1% osmic acid (Ted Pella) at room temperature in the dark for 2 h. After rinsing with PBS, the resin blocks were dehydrated, embedded, and polymerized. They were then sliced into ultra-thin sections measuring 60 nm using an ultra-thin slicing machine. Subsequently, the sections were stained with 2% uranyl acetate (SPI) and 2.6% lead citrate (Sigma) solutions for 8 min each. After rinsing, they were dried at room temperature overnight. Finally, they were photographed under a transmission electron microscope (TEM; HT7800, HITACHI, Japan).

### MDC staining assay

A total of 8 × 10^6^ RH tachyzoites, approximately twice the number of Vero cells, were added to a 75 cm^2^ cell culture flask. After 24 h, 5 μg/mL of GLA and 0.1% DMSO were added. Tachyzoites were purified after a 24-h compound incubation and then stained with 100 μM MDC (Sigma) for 1 h. After washing with PBS, a portion of the parasites from each group were observed under a laser confocal microscope, while the remaining portion was analyzed for fluorescence intensity using flow cytometry (CytoFLEX LX, Beckman, USA). Unstained tachyzoites were treated with 0.1% DMSO and served as the blank control. The results were analyzed using FlowJo™ v10.8 software (BD Life Sciences, USA).

### Mitochondria related assays

Relevant assays examining the effects of GLA on the mitochondria of *T. gondii* include measurements of reactive oxygen species (ROS), superoxide levels, and mitochondrial membrane potential. The preliminary experimental procedures for utilizing GLA to treat *T. gondii*-infected cells were consistent with the MDC staining assay. The distinction lies in the staining of purified tachyzoites with 100 nM MitoTracker™ red CMXRos (Thermo Fisher), 5 μM CM-HDCFDA (MCE), or 5 μM MitoSOX Red (MCE) at 37 °C for 30 min. After washing with PBS, the fluorescence intensity was measured using a flow cytometer, and data analysis was conducted using FlowJo™ v10.8 software.

### Statistical analysis

Statistical analyses were conducted using GraphPad Prism 9 software (San Diego, CA, USA). Results were expressed as mean ± standard error of the mean (SEM). Comparisons between two groups were performed using an independent samples *t*-test, while multigroup comparisons were performed using Tukey’s multiple comparison test following one-way analysis of variance. A *P*-value  < 0.05 was considered statistically significant.

## Results

### Screening of anti-*T. gondii* activity

**Fig. 1:**
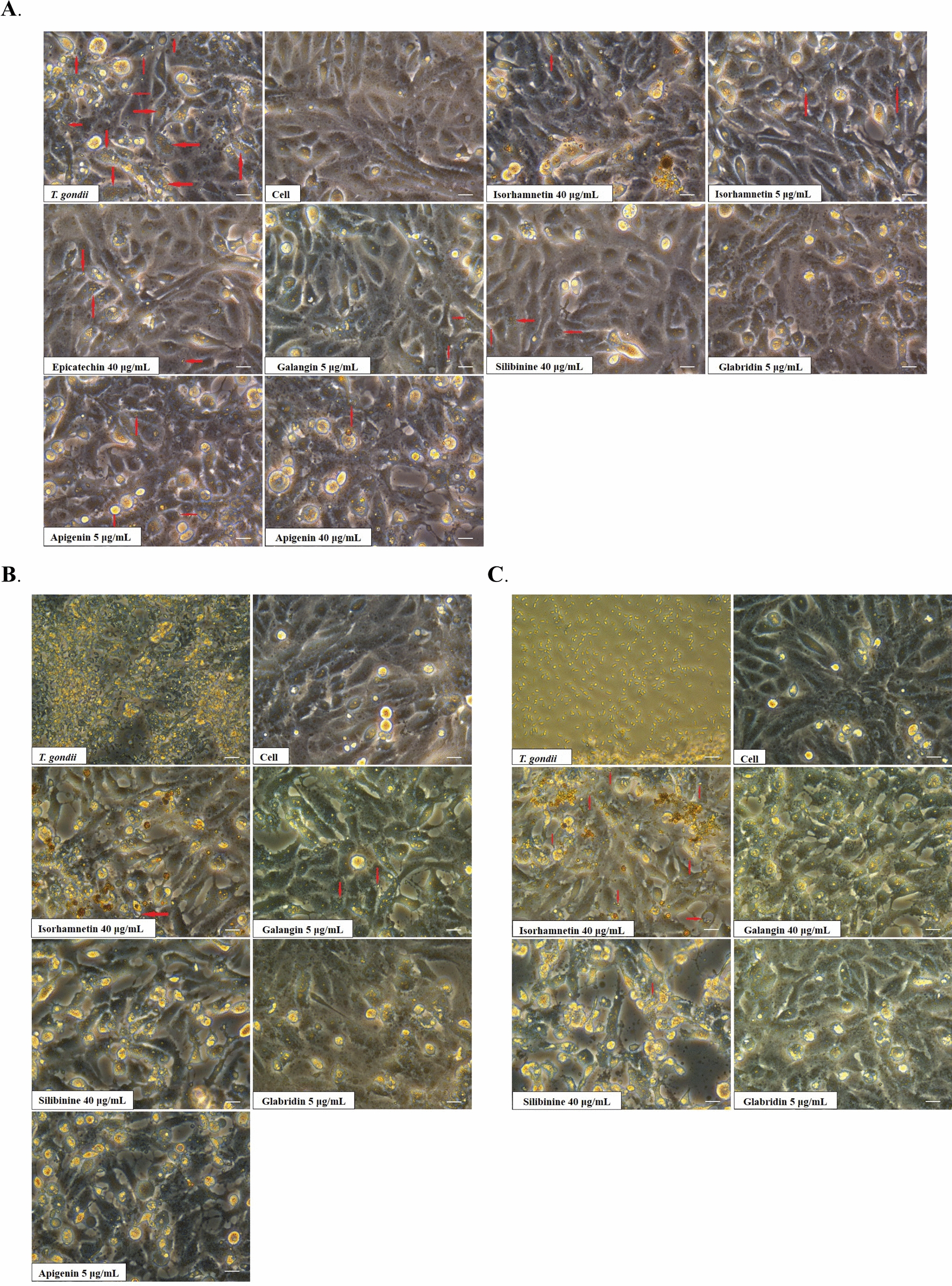
Effectiveness of flavonoids against *T. gondii*. After RH tachyzoites invasion of cells for 8 h, two concentrations of flavonoids were added: a high concentration (40 μg/mL) and a low concentration (5 μg/mL). The cells were then incubated for 24 h (**A**), 48 h (**B**), and 72 h (**C**). The red arrow represents *T. gondii*. Scale bar: 10 μm

In total, 20 flavonoids were initially screened at two concentrations: a high concentration of 40 μg/mL and a low concentration of 5 μg/mL. Compared with the control group, the number of *T. gondii* observed in the field of view was lowered by more than 80%, indicating significant effectiveness, as presented in Fig. 1 and Table 1. Isorhamnetin, apigenin, galangin, silibinin, and GLA demonstrated efficacy against *T. gondii*. However, isorhamnetin, apigenin, galangin, and silibinin exhibited cytotoxicity at effective concentrations. Although GLA displayed considerable toxicity to host cells at high concentrations, it did not exhibit significant toxicity at low concentrations and maintained pronounced activity against *T. gondii*, with no parasites detected at 24, 48, and 72 h (Fig. 1). Therefore, GLA was selected for further investigation.

### Cytotoxicity of GLA and its inhibitory effect on *T. gondii*

**Fig. 2:**
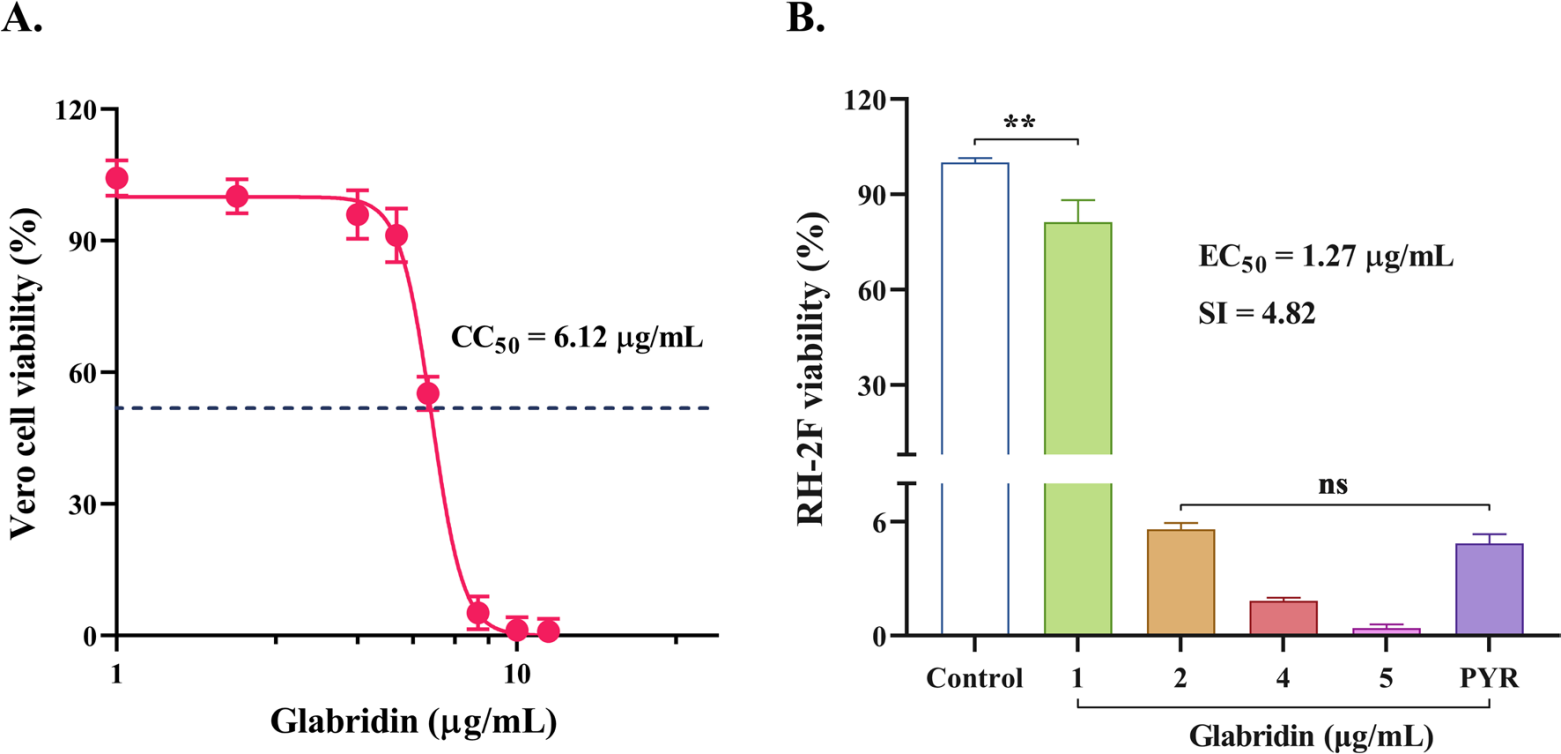
Inhibitory effects of glabridin on host cells and *T. gondii*. (**A**) Vero cell viability after treatment with glabridin for 72 h. CC_50_: The 50% cytotoxic concentration. (**B**) RH-2F viability after treatment with glabridin for 72 h. EC_50_, the 50% effective concentration; SI, selectivity index, defined as the ratio of CC_50_ to EC_50_. Reference drug: PYR-pyrimethamine. **, *P* < 0.01; ns, *P* > 0.05

The cytotoxicity of GLA on Vero cells was assessed over 72 h. As anticipated, toxicity exhibited a dose-dependent relationship, with a CC_50_ value of 6.12 μg/mL. At a concentration of 5 μg/mL, the cell survival rate was 91.24%. Thus, this concentration was selected as the maximum limit for the ensuing experiments (Fig. 2A). Following this, the inhibitory effect of GLA on *T. gondii* was evaluated. The results indicated that GLA significantly inhibited the growth of *T. gondii*. Specifically, 1 μg/mL of GLA markedly reduced the growth of the RH-2F strain. Furthermore, at a concentration of 2 μg/mL, GLA demonstrated an inhibitory effect comparable to that of the positive control drug PYR at 5 μg/mL. At the same time, the EC_50_ value of GLA against RH-2F was determined to be 1.27 μg/mL, with a selectivity index (SI) of 4.82 (Fig. 2B). In summary, our findings conjointly suggested that GLA exerts safe and effective inhibitory effects against *T. gondii*.

### Antiproliferative effect of GLA on *T. gondii*

**Fig. 3:**
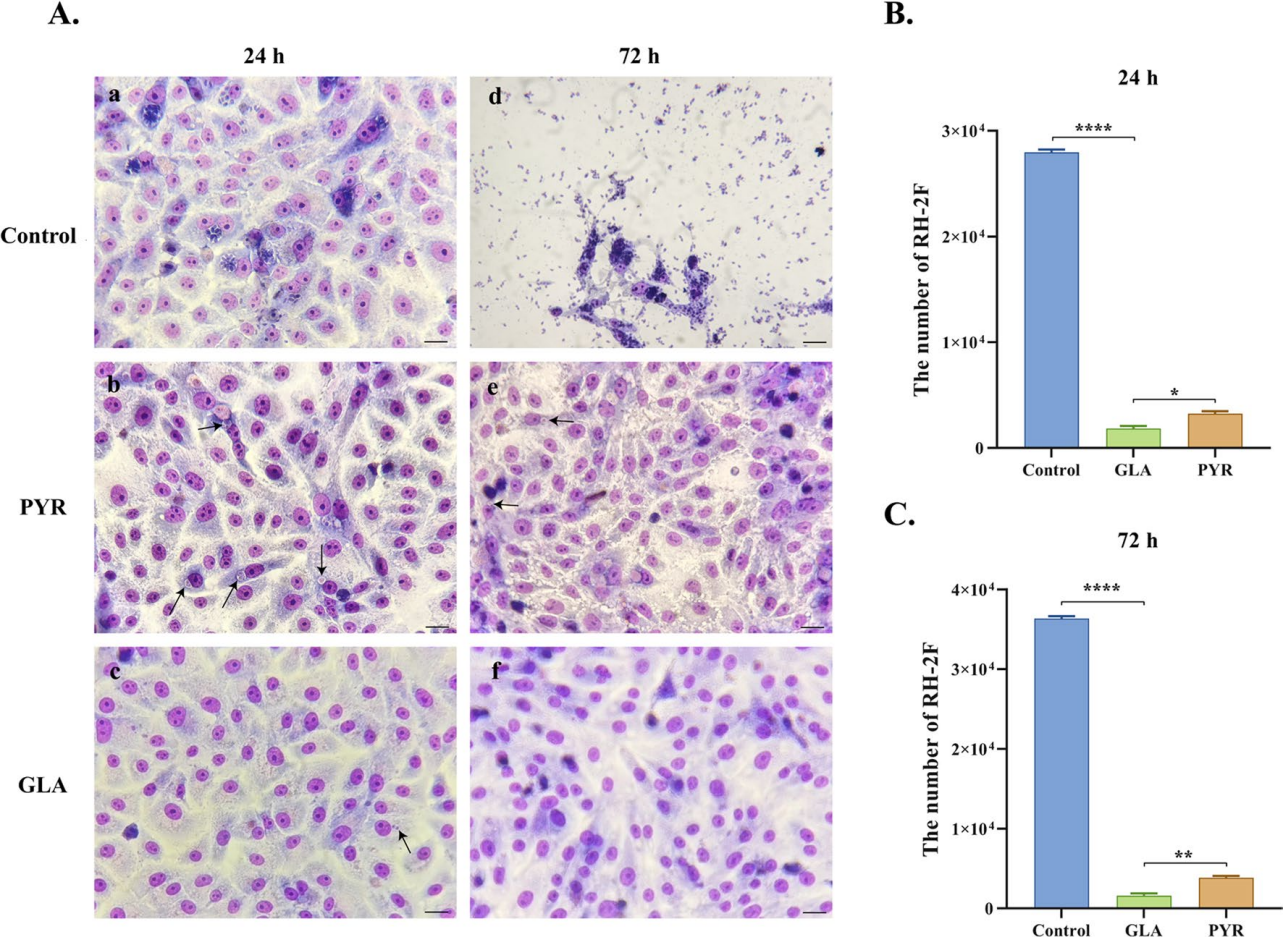
Effect of glabridin on the proliferation of *T. gondii*. (**A**) Giemsa staining of intracellular *T. gondii* at 24 h and 72 h post-treatment with GLA. Scale: 10 μm. (**B**) Quantification of *T. gondii* RH-2F 24 h after GLA treatment. (**C**) Quantification of *T. gondii* RH-2F 72 h after GLA treatment. GLA, glabridin. Reference drug: PYR-pyrimethamine. ****, *P* < 0.0001; **, *P* < 0.01; *, *P* < 0.05

In the Giemsa staining analysis, numerous individual tachyzoites and parasitophorous vacuoles were observed in the control group at 24 h (Fig. 3Aa), while only a markedly lower number of *T. gondii* were present in the GLA group (Fig. 3Ac). By 72 h, the cellular structure in the control group had been largely compromised by the parasites, with the cytoplasm of the few remaining cells also filled with parasites (Fig. 3Ad). In contrast, virtually no parasites were identified in the GLA group (Fig. 3Af), while a small number of parasites was detected in the PYR group (Fig. 3Ae). The number of intracellular parasites was quantified at both 24 and 72 h, and the results were consistent with those obtained from Giemsa staining. Taken together, these findings indicate that GLA can significantly inhibit the proliferation of intracellular *T. gondii*, thereby exerting antiproliferative effects that are significantly superior to those of PYR (Fig. 3B and C).

### Effect of GLA on the lysis cycle and invasion of *T. gondii*

**Fig. 4:**
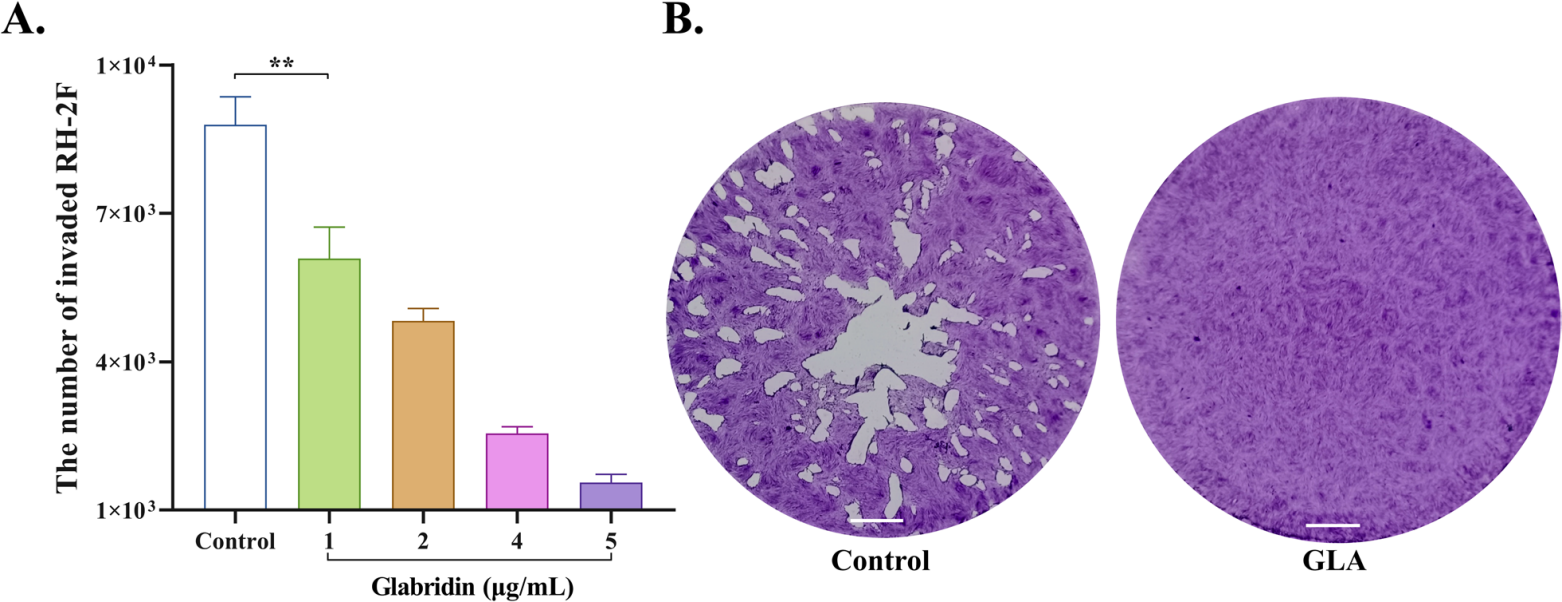
Glabridin influences the invasion and lytic cycle of *T. gondii*. (**A**) Effect of GLA on the invasive capabilities of *T. gondii* into host cells within the first 2 h. (**B**) Effect of GLA on *T. gondii* plaque formation in host cells. GLA, glabridin. Scale: 2 mm

The ability of *T. gondii* tachyzoites to invade host cells was evaluated after 2 h of treatment with GLA. The results signaled that GLA exerted significant anti-invasive effects on RH-2F in a dose-dependent manner. Specifically, a concentration of 1 μg/mL of GLA markedly inhibited the invasion of host cells by *T. gondii* (Fig. 4A). To further elucidate the effects of GLA on the entire lytic cycle of *T. gondii*, a plaque assay was carried out. Our findings revealed that, in contrast to the abundant plaques observed in the control group, no plaques were visualized in the GLA group (Fig. 4B), suggesting that GLA disrupts the lytic cycle of *T. gondii*.

### Effect of GLA on bradyzoites and cysts of *T. gondii*

**Fig. 5:**
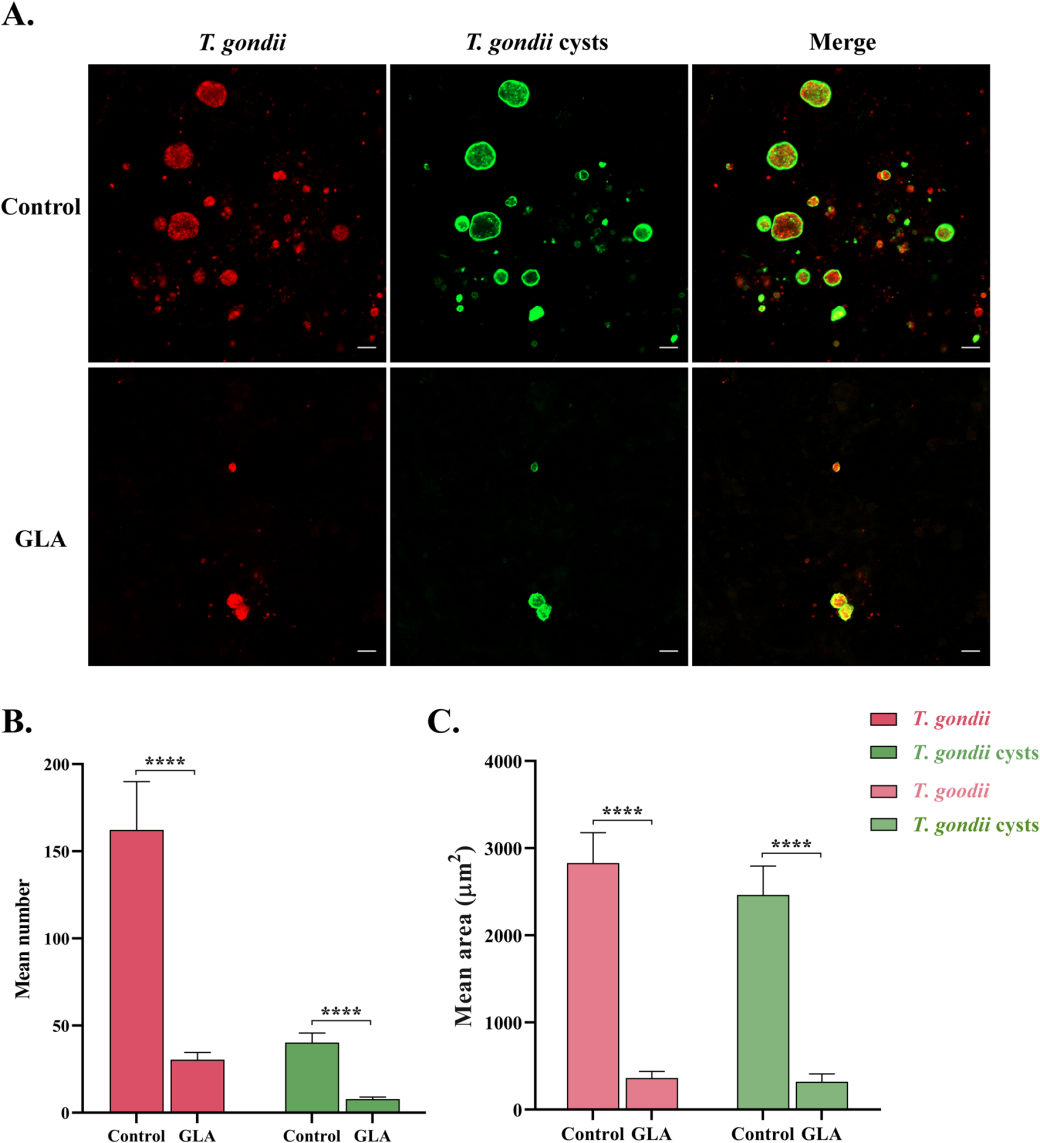
Glabridin is effective for *T. gondii* bradyzoites in vitro. Effect of GLA on *T. gondii* bradyzoites differentiation in vitro over 7 days. *T. gondii* was labeled with specific antibodies (red fluorescence), while cysts were stained with FITC-DBA (green fluorescence). Photographs were captured under a laser confocal microscope (**A**), and ImageJ software was utilized to calculate the mean number (**B**) and mean area (**C**) of *T. gondii* and cysts across 15 images. GLA, glabridin. Scale: 10 μm

Given that GLA exerts significant effects on *T. gondii* tachyzoites in vitro, its effects on the bradyzoites and cysts of *T. gondii* were explored. Interestingly, our findings suggested that GLA can significantly reduce the number of *T. gondii* bradyzoites and cysts (Fig. 5A, B). Although GLA did not completely eliminate cysts from the cells, it markedly restricted their growth. Notably, the size of the cysts was significantly smaller compared with the control group (Fig. 5A, C).

### Effect of GLA on the ultrastructure of *T. gondii*

**Fig. 6:**
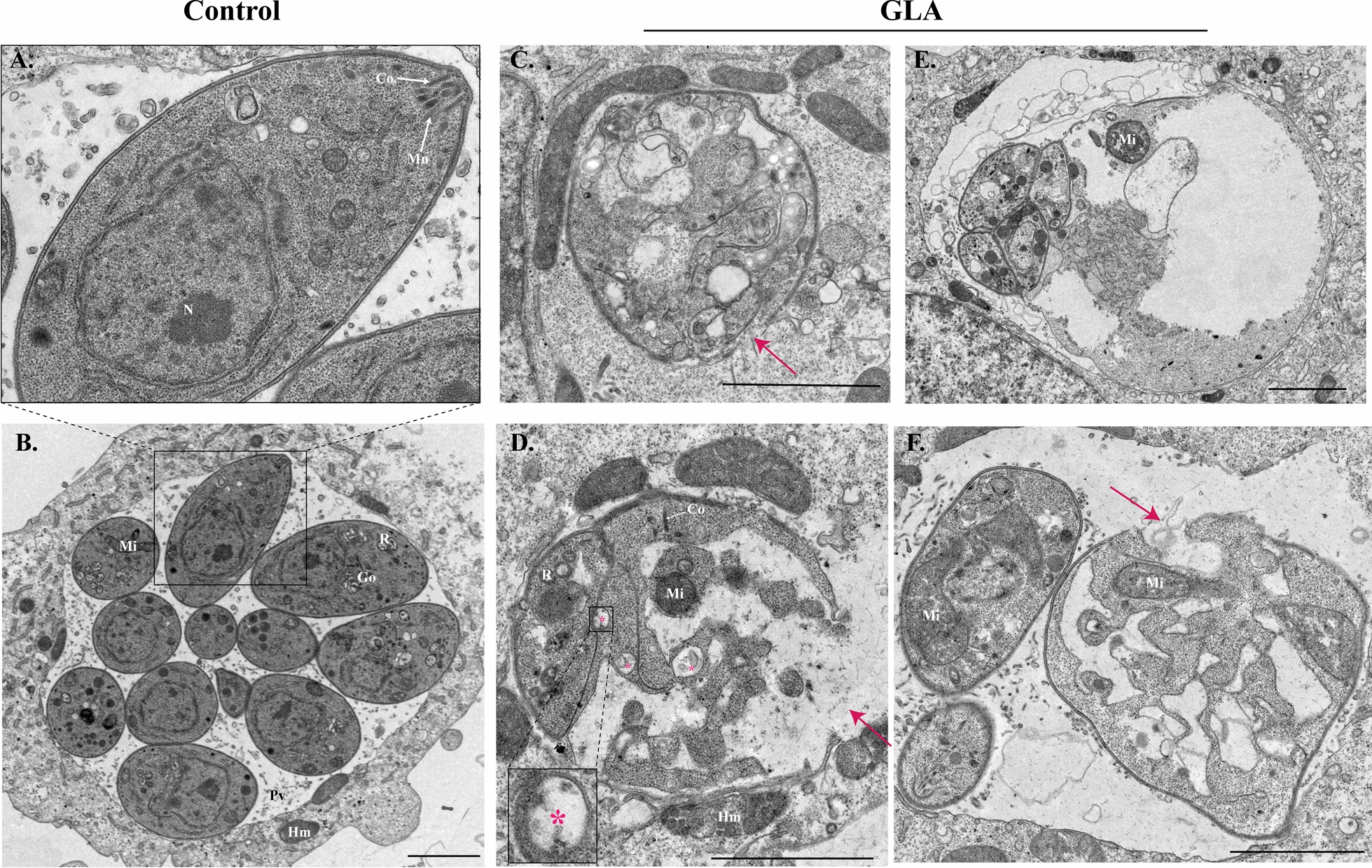
Effect of glabridin on the ultrastructure of *T. gondii.* (**A**) and (**B**) Ultrastructure of *T. gondii* in the control group. (**C**), (**D**), (**E**), and (**F**) Ultrastructure of *T. gondii* after 24 h of GLA treatment. The asterisk indicates the autophagic lysosome and the red arrow points to the ruptured cell membrane. Co, conoid; Hm, host mitochondria; Mn, micronemes; N, nucleus; PV, parasitophorous vacuole; R, rhoptry; Mi, mitochondria; GLA, glabridin. Scale: 2 μm

Furthermore, the effects of GLA on the ultrastructure of *T. gondii* were investigated using TEM. In the control group, *T. gondii* proliferated within host cells, with the typical structures of organelles, including rhoptry, nucleus, conoid, micronemes, and mitochondria, being well defined (Fig. 6A, B). However, degradation and disappearance of various organelles were noted in *T. gondii* treated with GLA (Fig. 6C). Of note, mitochondria appeared to be the last organelles to disappear (Fig. 6D–F). While some mitochondria were swollen and exhibited a loss of cristae (Fig. 5F), others remained intact (Fig. 6D). Additionally, the size of several *T. gondii* was increased, accompanied by cell membrane rupture (red arrows) and the presence of autolysosomes (asterisks) (Fig. 6).

### Effect of GLA on autophagy of *T. gondii*

**Fig. 7:**
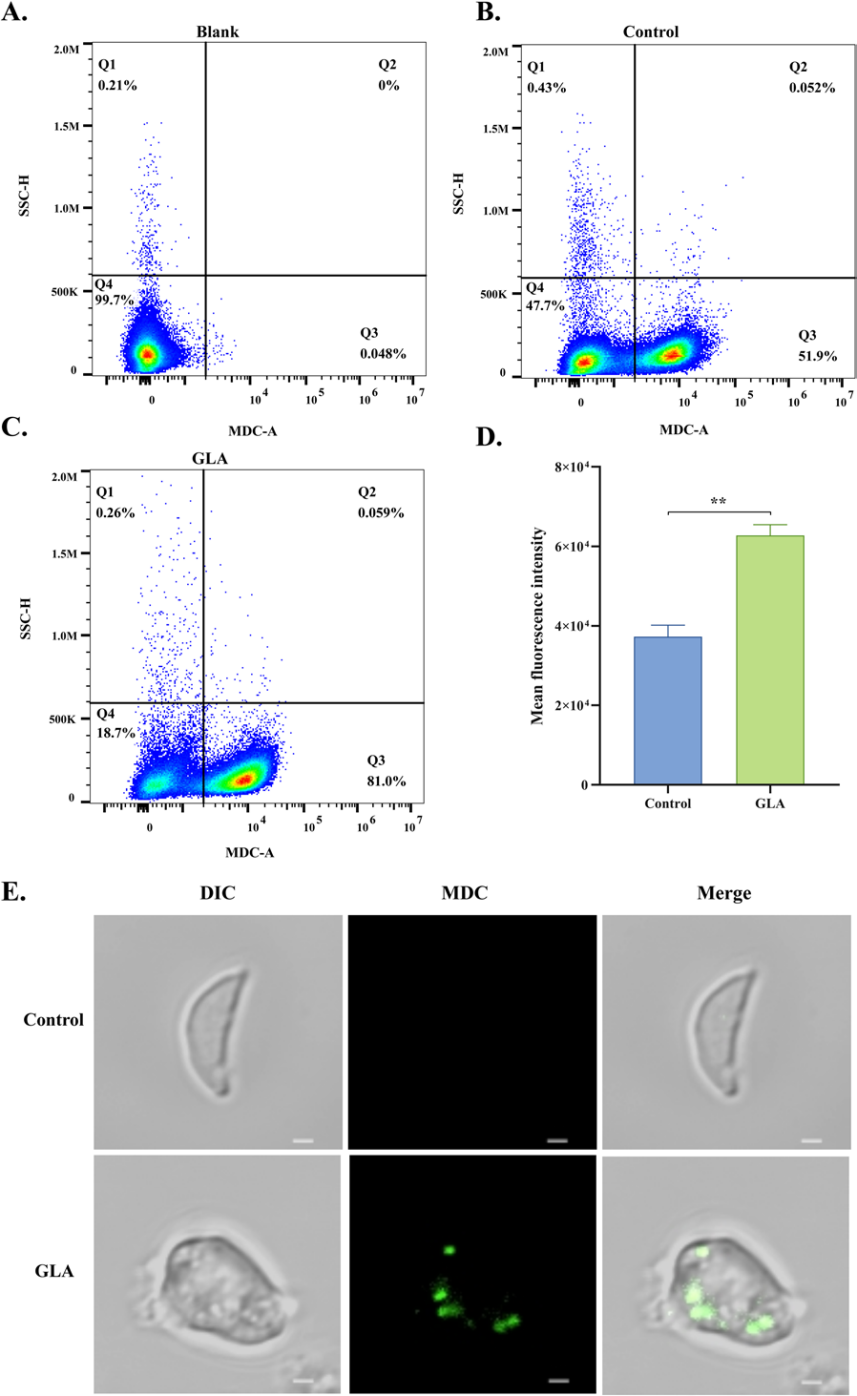
Glabridin treatment induces autophagy in *T. gondii*. A total of 24 h after GLA treatment, *T. gondii* was stained with MDC and analyzed using flow cytometry. (**A**) displays the flow cytometry chart for *T. gondii* without MDC staining, serving as a blank control group. (**B**) presents the flow cytometry chart for the 0.1% DMSO treatment. (**C**) illustrates the flow cytometry chart for the GLA treatment. (**D**) depicts the mean fluorescence intensity of *T. gondii* MDC staining in the control and GLA groups, calculated using FlowJo™ v10.8 software. (**E**) presents images captured under a confocal laser microscope following MDC staining. Monodansylcadaverine, MDC; GLA, glabridin. Scale: 1 μm. **, *P* < 0.01

Under TEM, autophagic lysosomes were visible, implying that GLA may induce autophagy in *T. gondii*. To validate this hypothesis, *T. gondii* was stained with MDC and assessed using flow cytometry. The results demonstrated that 24 h after GLA treatment, 81% of tachyzoites were stained with MDC, significantly higher than the 51.9% observed in the control group (Fig. 7B, C). Furthermore, the average fluorescence intensity was calculated for both groups, and it revealed that the average fluorescence intensity was significantly higher in the GLA group compared with the control group (Fig. 7D). Finally, laser confocal microscopy of *T. gondii* delineated that tachyzoites in the control group maintained their typical crescent-shaped structure, while several tachyzoites in the GLA group displayed abnormal morphologies. This observation was in line with the findings from TEM, where expanded subunit volumes, ruptured membranes, and the presence of autophagic lysosomes were noted (Fig. 7E). Overall, our results indicate that GLA can effectively induce autophagy in *T. gondii*.

### Effect of GLA on mitochondria of *T. gondii*

**Fig. 8:**
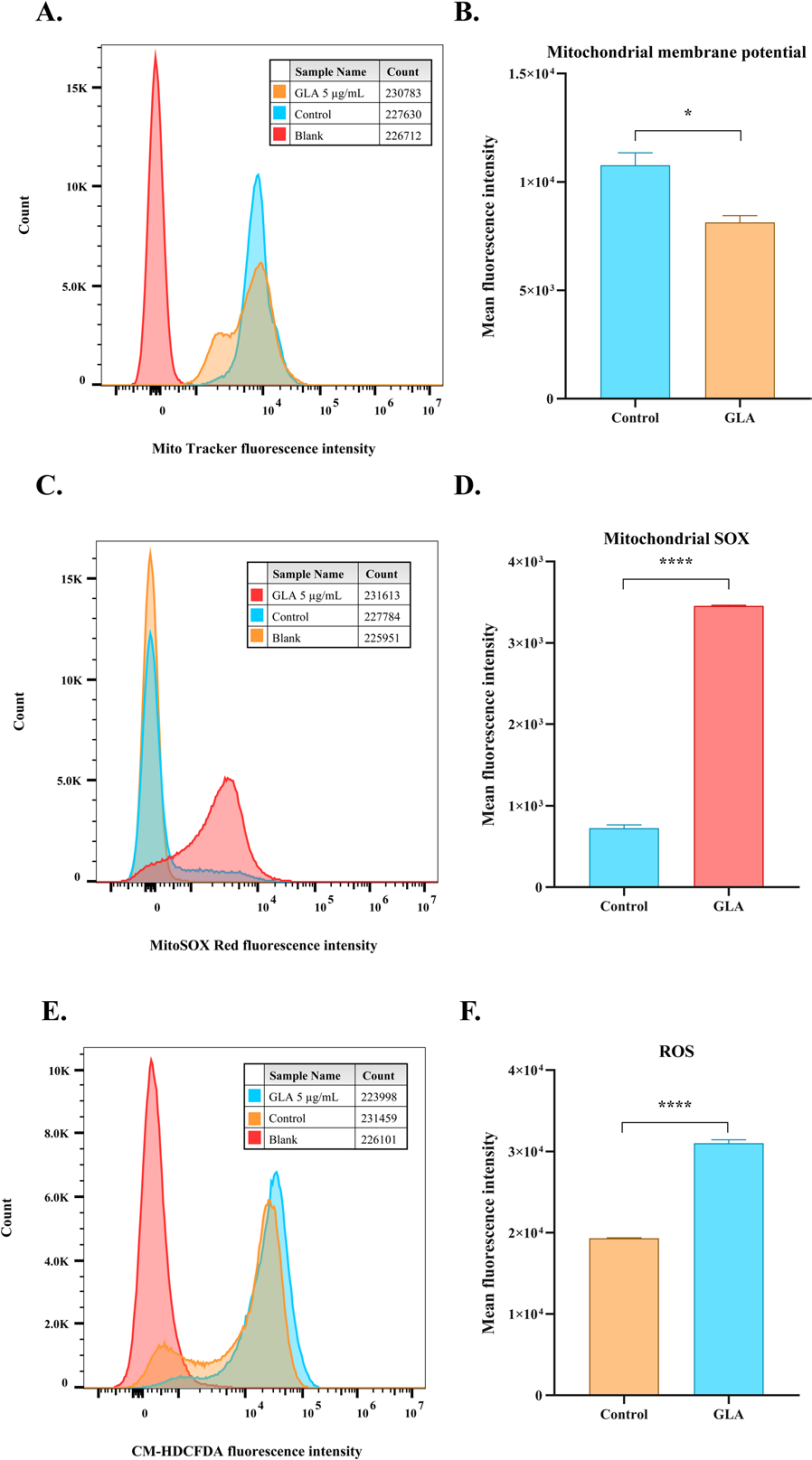
Effect of glabridin on *T. gondii*’s mitochondrial activity. GLA was applied to intracellular *T. gondii* for 24 h, followed by staining with the fluorescent probes MitoTracker™ Red CMXRos, CM-HDCFDA, or MitoSOX Red, and analyzed using a flow cytometer. (**A**), (**C**), and (**E**) display the flow cytometry charts of *T. gondii* stained with each fluorescent probe. (**B**), (**D**), and (**F**) present the mean fluorescence intensity of *T. gondii* staining in the control and GLA groups, calculated using FlowJo™ v10.8 software. *, *P* < 0.05; ****, *P* < 0.0001

TEM revealed that the mitochondria of some *T. gondii* were swollen following GLA treatment, suggesting that GLA may induce mitochondrial dysfunction in *T. gondii*. Consequently, we investigated the effects of GLA on mitochondrial membrane potential, superoxide levels, and reactive oxygen species (ROS) levels in *T. gondii*. Our results demonstrated that GLA treatment of intracellular *T. gondii* for 24 h significantly reduced the mitochondrial membrane potential (Fig. 8A, B), increased the levels of superoxide in T. gondii mitochondria (Fig. 8C, D), and elevated the levels of reactive oxygen species in T. gondii (Fig. 8E, F). These findings indicate that GLA can induce mitochondrial dysfunction in *T. gondii*.

## Discussion

As is well documented, toxoplasmosis is a significant zoonotic parasitic disease [21]. Currently, there are no effective preventive measures and no treatment methods for eradicating *T. gondii* infection. Existing therapeutic drugs are associated with numerous adverse reactions, and resistance to these drugs has been documented [22]. Therefore, there is an urgent need to pioneer new therapies targeting *T. gondii* infections. In this study, 20 flavonoids were selected to screen for their anti-*T. gondii* activity. To the best of our knowledge, this is the first study to investigate the effects of GLA on both the tachyzoite and bradyzoite stages of *T. gondii *in vitro.

GLA is a prenylated isoflavone compound derived from licorice root, recognized for its various biological properties, including antioxidant, antiinflammatory, anti-atherosclerotic, and skin-whitening activities [23]. This study demonstrated that GLA exhibited significant activity against both the tachyzoite and bradyzoite stages of *T. gondii *in vitro. In addition, GLA exerted significant inhibitory, anti-invasive, and antiproliferative effects on *T. gondii*, thereby disrupting its lytic cycle. Of note, at the same concentration, the antiproliferative effect of GLA was significantly superior to that of PYR (Fig. 3). Furthermore, GLA displayed significant activity against the bradyzoites and cysts of *T. gondii*, leading to a marked reduction in both the number and size of *T. gondii* cysts (Fig. 5). Additionally, earlier studies have reported that GLA exerts neuroprotective effects, can reverse learning and memory deficits in diabetic rats, and possesses sedative and anxiolytic properties [24, 25]. These findings signal that GLA may be effective against chronic toxoplasmosis. Nevertheless, further studies are necessitated to validate this hypothesis.

In this study, transmission electron microscopy, laser confocal microscopy, and flow cytometry were used to elucidate the mode of action of GLA on *T. gondii*. The findings indicated that GLA induced an increase in reactive oxygen species, a decrease in mitochondrial membrane potential, and an elevation in mitochondrial superoxide levels, along with autophagy and membrane rupture of *T. gondii*. Previous studies have established that the antibacterial mechanism of GLA against *Listeria monocytogenes* involves its action on the cell membrane, resulting in membrane penetration and structural destruction [26]. Additionally, Yang et al. concluded that the bactericidal action of GLA against *Fusarium graminearum* is characterized by the disruption of cell membrane integrity, leading to disturbances in intracellular substances and energy metabolism [27]. These findings suggest that GLA may also influence *T. gondii* by altering the permeability of its cell membrane and inducing membrane rupture; however, the specific mode of action warrants further investigation.

## Conclusions

Flavonoids demonstrate safe and effective inhibitory effects on *T. gondii *in vitro, exerting significant anti-invasive and antiproliferative effects that disrupt the lytic cycle of the parasite. It is worthwhile to emphasize that GLA exerts a pronounced inhibitory effect on both the bradyzoites and cysts of *T. gondii*, leading to a significant reduction in both the number and size of cysts. Finally, our results suggested that GLA’s mechanism of action may entail the induction of mitochondrial dysfunction, autophagy, and membrane rupture in *T. gondii*.

## Data Availability

No datasets were generated or analyzed during the current study.

## Abbreviations

*T. gondii*: *Toxoplasma gondii*
RH-2F: *T. gondii* RH tachyzoites expressing β-galactosidasde
DMEM: Dulbecco’s modified eagle medium
FBS: Fetal bovine serum
DMSO: Dimethyl sulfoxide
PBS: Phosphate buffer solution
CCK-8: Cell counting kit 8
GLA: Glabridin
PRU: Prugniaud
TEM: Transmission electron microscopy
MDC: Monodansylcadaverine
PYR: Pyrimethamine
SEM: Standard error of mean SI
SI: Selectivity index
CPRG: Chlorophenol red-β-D-galactopyranoside
CC_50_: The 50% cytotoxic concentration
EC_50_: The 50% effective concentration
Vero: African green monkey kidney
HFF: Human foreskin fibroblasts
ROS: Reactive oxygen species
SOX: Superoxide
